# Geographic variation and allometry shape head morphology more than diet in the Mexican black-bellied garter snake (*Thamnophis melanogaster*)

**DOI:** 10.7717/peerj.21624

**Published:** 2026-08-06

**Authors:** Javier Manjarrez, Armando Sunny, Felipe de Jesús Rodríguez-Romero

**Affiliations:** 1 Facultad de Ciencias, Universidad Autónoma del Estado de México, Toluca, Estado de Mexico, Mexico; 2 Centro de Investigación en Ciencias Biológicas Aplicadas, Universidad Autónoma del Estado de México, Toluca, Estado de Mexico, Mexico; 3 Facultad de Ciencias, Universidad Autónoma del Estado de México, Toluca, Estado de México, Mexico

**Keywords:** Geometric morphometrics, Piscivory, Phenotypic variation, Morphological evolution, Trophic ecology, Procrustes analysis, PCA, Morphospace

## Abstract

In snakes, head morphology is closely linked to feeding ecology, particularly in aquatic systems where prey capture imposes strong functional constraints. Here, we examined head shape variation in the Mexican black-bellied garter snake (*Thamnophis melanogaster*) across four populations with contrasting diets (piscivorous *vs.* non-piscivorous) using geometric and linear morphometric approaches. Contrary to our initial hypothesis, geometric morphometric analyses revealed that head shape variation is primarily structured by geographic differences among populations rather than diet. Significant differences in head shape were detected among localities (*p* < 0.001), with Chapala representing the most distinct population, while no significant effect of sex was detected. Allometry had a significant effect on head shape, but the allometric trajectories were consistent across populations. In contrast, linear morphometric analyses showed that although head size scales similarly to body size across dietary groups, relative head size differs significantly between piscivorous and non-piscivorous individuals (*p* < 0.01), with non-piscivorous snakes exhibiting proportionally larger heads. These results indicate that diet does not drive major differences in head shape but does influence relative head proportions. Overall, our findings highlight the complex interplay between geography, allometry, and diet in shaping morphological variation in *T. melanogaster*, suggesting that multiple ecological factors beyond prey type contribute to head morphology in this species.

## Introduction

Because obtaining food resources is essential for survival, the diet and morphological phenotype of snakes are closely linked (Herrel et al., 2008; Mori & Vincent, 2008; Moon et al., 2019). Prey characteristics can induce morphological differences that often reflect phenotypic plasticity rather than underlying genetic divergence (Krause, Burghardt & Gillingham, 2003; Klaczko, Sherratt & Setz, 2016; Perkins & Eason, 2019). Such plastic responses in feeding-related morphology have been widely documented across animal taxa, frequently in relation to prey size, shape, defenses, and other ecological attributes (Madsen & Shine, 1993; Clifton, Chamberlain & Gifford, 2017; Hudry & Herrel, 2025).

Snakes represent an ideal system for studying the relationship between morphology and feeding ecology because, as gape-limited predators, the head plays a central role in prey capture and ingestion (Mori & Vincent, 2008; Fabre et al., 2016; Moon et al., 2019; Deepak, Gower & Cooper, 2023). Since snakes consume their prey whole, head dimensions and cranial structure are expected to be closely associated with prey characteristics such as size, shape, and consistency (Mori & Vincent, 2008; Vincent et al., 2009; Hampton, 2014). Indeed, several studies have reported direct relationships between head morphology and diet in snakes (Vincent & Herrel, 2007; Hampton, 2014; Clifton, Chamberlain & Gifford, 2017). In addition to diet, intraspecific geographic variation has been recognized as an important source of morphological diversity in snakes (Queral-Regil & King, 1998; Allsteadt et al., 2006). Variation in head dimensions among populations may reflect phenotypic plasticity in response to feeding experience but may also be driven by broader ecological differences among habitats (Queral-Regil & King, 1998). For example, natricine snakes consume a wide range of soft-bodied prey, including earthworms, amphibians, and fish (Rossman, Ford & Seigel, 1996; Gibbons & Dorcas, 2004; Hudry & Herrel, 2025), and head morphology in these taxa has been linked to feeding specialization (Mori & Vincent, 2008; Vincent et al., 2009; Hampton, 2011; Hampton, 2014). In particular, elongated and slender head shapes have been proposed as adaptations for consuming soft or elongated prey such as fish or slugs (Herrel et al., 2008; Vincent et al., 2009; Hudry & Herrel, 2025). Piscivory, in particular, imposes distinct functional demands compared to feeding on amphibians or other prey types. Capturing fish in aquatic environments requires overcoming hydrodynamic constraints in a dense and viscous medium, which may influence the evolution of cranial morphology (Alfaro, 2002; Vincent et al., 2009; Segall et al., 2016). For instance, piscivorous snakes often exhibit morphological traits such as elongated skull elements or modified quadrate orientations, which are thought to enhance prey capture efficiency and ingestion performance (Dwyer & Kaiser, 1997; Vincent et al., 2009). Similarly, increases in head length or related structures may be associated with greater gape and improved prey handling capabilities (Vincent et al., 2009). These patterns suggest that diet, particularly piscivory, could play a significant role in shaping head morphology. Sexual dimorphism is another factor frequently associated with variation in head morphology in snakes. In many species, females tend to have relatively larger heads than males do, which has been linked to differences in prey size and feeding ecology (Shine, 1991; Shine, 1994a; Vincent & Herrel, 2007; Meik et al., 2012). However, the extent to which sexual dimorphism interacts with diet and geographic variation in shaping head morphology remains unclear and may vary among species. Although diet has traditionally been considered a primary driver of morphological variation, populations inhabiting different environments often exhibit distinct morphologies that reflect a combination of ecological factors, including habitat structure, resource availability, and predator–prey interactions (Marko, 2005; Rivera, 2008). Consequently, disentangling the relative contributions of diet and geographic variation is essential for understanding the drivers of morphological diversification.

In this study, we examined head morphology in the Mexican black-bellied garter snake (*Thamnophis melanogaster*), a species that exhibits marked geographic variation in diet. Some populations are predominantly piscivorous, whereas others consume a broader range of prey, including amphibians, leeches, and crayfish (Manjarrez, Macías-Garcia & Drummond, 2013; Manjarrez, Macías-Garcia & Drummond, 2017). Previous work has demonstrated that this species can exhibit morphological plasticity in response to dietary shifts, particularly in relation to novel prey such as crayfish (Manjarrez, Macías-Garcia & Drummond, 2017; Manjarrez, Macias-Garcia & Drummond, 2020), making it an ideal system for evaluating the relationship between diet and morphology. To disentangle the relative roles of diet, geography, and sex in shaping head morphology, we analyzed the variation in head shape and size across four populations of *T. melanogaster* with contrasting diets (piscivorous *vs.* non-piscivorous). Specifically, we tested whether head morphology is primarily structured by dietary differences or by geographic variation among populations. We predicted that, if diet is the dominant factor, piscivorous populations would exhibit distinct head shapes compared to non-piscivorous populations. Alternatively, if geographic factors play a stronger role, morphological variation would be structured primarily by locality rather than diet.

## Materials and Methods

Snakes were collected between 1981 and 2001 during previous studies on the ecology of snakes in central Mexico (Manjarrez, Macias-García & Drummond, 2013; Manjarrez, Macías-Garcia & Drummond, 2017; Manjarrez, Macias-Garcia & Drummond, 2020; Manjarrez, Pacheco-Tinoco & Venegas-Barrera, 2017; González-Fernández et al., 2018; Valencia-Flores et al., 2019). Two piscivore locations and two nonpiscivore locations were classified according to the criteria of prey availability and diet recorded over three decades (Manjarrez, Macias-García & Drummond, 2017). The piscivore sites were Lake Chapala (100% of snakes consume fish; altitude of 1,524 masl; 20°13′50″N, 103°02′41″W) in Jalisco State and Cuitzeo Lake (98% of snakes consume fish; Manjarrez, Macias-García & Drummond, 2013; Manjarrez, Macias-Garcia & Drummond, 2020; altitude of 1,840 masl; 19°56′00″N, 101°05′00″W) in Michoacan State. The nonpiscivore sites were in Mexican State, San Pedro (altitude of 2,575 m, 19°11′N, 99°31′W), and Acambay (altitude of 2,560 m, 19°55′N, 99°49′W; INEGI, 2013). In both populations, *T. melanogaster* consumes tadpoles, leeches, and crayfish; 0% of these individuals consume the fish (Manjarrez, Macias-García & Drummond, 2013; Manjarrez, Macias-Garcia & Drummond, 2020).

All the snakes were collected under a scientific collection permit issued by the federal institution (Secretaría de Medio Ambiente y Recursos Naturales, SCPA/DCVS/02685/19) and the ethics committee of the Universidad Autónoma del Estado de México (4047/2016SF, 4865/2019SF). All the subjects were treated humanely in accordance with the guidelines of the Mexican Federal Regulation for Animal Experimentation and Care (NOM-062-ZOO-2001) and the guidelines outlined by the American Society of Ichthyologists and Herpetologists (ASIH, 2004).

At these sites, the snakes were manually captured and measured (snout-vent length, SVL) and sexed by visual inspection of the tail-base thickness or by manually everting the hemipenis in small snakes. In the laboratory, the snakes were housed, and their head dimensions were estimated as described later. Our study included 31 adult males and 72 adult females. The difference in sample size between males and females arises from the time period of the study, which included the months when gravid females thermoregulate on the ground and are found more commonly than males.

## Geometric Morphometric Data

Images were obtained by taking dorsal photographs of the snakes’ heads with a SONY 14.2-megapixel camera and a 2.8/50 macrolens, all taken at a distance of 15 cm from the snake’s head. A sheet of graph paper was placed under the snakes’ heads to serve as a background for the resulting image, providing a specific unit of measurement for reference. All the images obtained were saved in JPG format and then organized into files according to location and sex to obtain intrapopulation results. To compare all the locations, a new file containing all the images from each location was created. Initial landmark configurations were aligned using the IMP (Nair, 2016 package); however, all subsequent geometric morphometric analyses were conducted in R (R Core Team, 2024), and landmark configurations were aligned to remove the effects of position, scale, and orientation by performing a series of displacements, size adjustments, and algebraic rotations. The morphotypes of males and females from the analyzed localities were obtained using thin plate spline analysis with the PCAGen6p module of IMP. To observe the differences in shape, we used deformation grids based on average reference point configurations for males and females.

**Figure 1:**
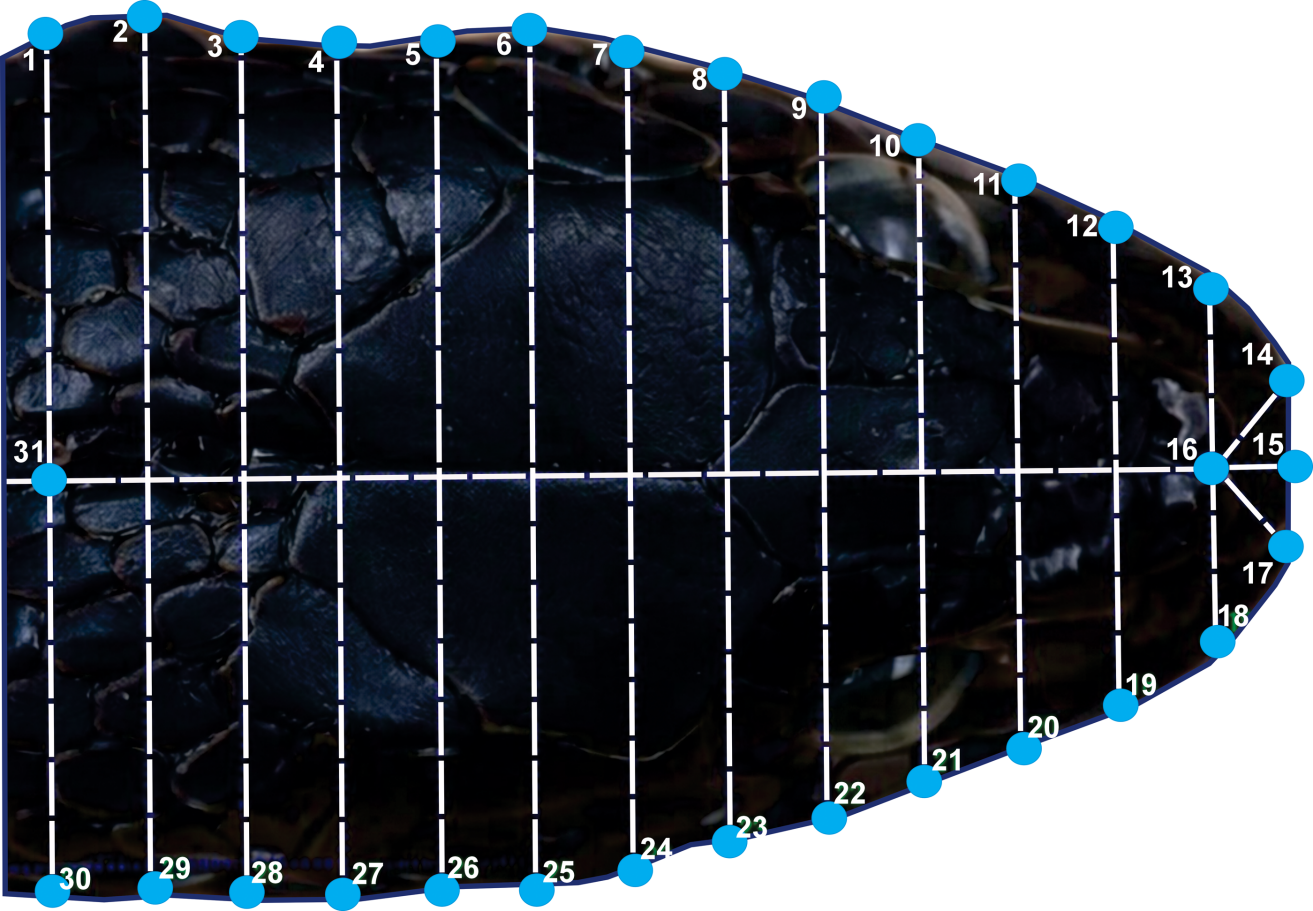
Landmark configuration used for geometric morphometric analysis of head shape in *Thamnophis melanogaster*. A total of 31 homologous landmarks were digitized on the dorsal view of the head to capture variation in snout shape, cranial width, and posterior head structure. Landmarks represent anatomically consistent points across individuals and were used to quantify shape variation following Procrustes superimposition.

Head shape variation was analyzed using geometric morphometric techniques based on a set of homologous landmarks distributed across the dorsal surface of the head. A total of 31 landmarks were defined to capture variations in snout shape, cranial width, and posterior head configuration (Fig. 1). Landmarks were digitized using TPSDig2, and coordinate data were exported in TPS format and subsequently imported into R for analysis. All geometric morphometric analyses were conducted in R using the package geomorph (Collyer & Adams, 2018; Baken et al., 2021; Collyer & Adams, 2024; Adams et al., 2026), complemented with dplyr, tidyr, ggplot2, gstatsplot, car, and base stats functions (Wickham, 2016; Fox & Weisberg, 2019; Patil, 2021; Wickham, Vaughan & Girlich, 2025; Wickham et al., 2026). Landmark configurations were subjected to generalized procrustes analysis (GPA) to remove nonshape variation associated with translation, rotation, and scale while preserving shape information. Centroid size was retained as a measure of overall head size and was considered in subsequent analyses to evaluate potential allometric effects. To explore patterns of morphological variation, a principal component analysis (PCA) was performed on Procrustes-aligned coordinates. Principal component scores were extracted and used to visualize morphospace occupation across localities (Acambay, Chapala, Cuitzeo, and San Pedro) and between sexes. Confidence ellipses (95%) were incorporated to represent group dispersion in morphospace. Shape variation associated with principal components was visualized using thin-plate spline deformation grids and wireframe plots, which represent mean shapes derived from Procrustes-aligned coordinates. Prior to statistical comparisons, assumptions of normality and homogeneity of variance were evaluated using Shapiro–Wilk tests implemented in stats and Levene’s tests using the car package. Because the PC1 scores did not meet parametric assumptions, nonparametric analyses were employed. Differences in head shape among localities were assessed using a Kruskal–Wallis test, followed by pairwise Wilcoxon rank-sum tests with Benjamini–Hochberg (BH) correction for multiple comparisons. These comparisons were visualized using the ggstatsplot package, specifically through functions such as ggbetweenstats, which integrate violin plots, boxplots, effect sizes, and adjusted *p* values within a single graphical framework. Additionally, a Procrustes analysis of variance (ANOVA) was performed using the geomorph package to evaluate the effects of locality, sex, and their interaction on head shape. Statistical significance was assessed using permutation procedures (999 iterations), and effect sizes were calculated to quantify the relative contribution of each factor. Morphological differentiation among localities was further explored by calculating pairwise Procrustes distances among mean shapes. These distances were visualized using hierarchical clustering (Ward method, hclust) and heatmaps generated in ggplot2, allowing identification of similarity patterns among populations. Morphological trajectories among populations were reconstructed based on mean principal component scores and visualized in morphospace (Adams et al., 2021) for R. Additionally, kernel density distributions of PC1 scores were generated using ggplot2 to compare the distribution of shape variation across localities.

### Linear morphometrics

Linear morphometric analyses were also conducted to evaluate differences in head size and relative proportions between piscivorous and non-piscivorous individuals. The morphometric variables included snout–vent length (SVL; hereafter referred to as LHC), head length (HL), head width (HW), and body mass (BM). The data were imported using the readr (Wickham et al., 2026) package and processed using dplyr, including standardization of variable names and merging of datasets from piscivorous and non-piscivorous populations. Records with missing values for key variables were excluded prior to analysis. To assess the allometric relationships between head size and body size, linear models were fitted using ordinary least squares (OLS) implemented in the stats package. Specifically, head length (HL) was modeled as a function of body size (LHC) and diet category (diet), including their interaction term (HL∼LHC * diet), to evaluate whether scaling relationships differed between piscivorous and non-piscivorous individuals. Model assumptions were evaluated prior to inference. The normality of the residuals was assessed using the Shapiro–Wilk test in the stats package for R, and the homogeneity of variance was evaluated using Levene’s test implemented in the car package. Additionally, diagnostic plots (residuals *vs.* fitted values and Q–Q plots) were inspected. Because the residuals did not fully meet the parametric assumptions, complementary nonparametric analyses were also conducted. To obtain size-corrected measures of head size, residuals were extracted from a reduced linear model (HL∼LHC) fitted using stats for R. These residuals (residual head size) were interpreted as relative head size independent of body size. Differences in residual head size between dietary groups were evaluated using the Mann–Whitney U test (Wilcoxon rank-sum test) implemented in stats. In addition, a proportional index of head size was calculated as the ratio of head length to body size (HL/LHC), using dplyr for data transformation. Differences between piscivorous and non-piscivorous individuals in this ratio were also evaluated using a Mann–Whitney U test implemented in stats for R. Allometric relationships were visualized using scatterplots with fitted regression lines and 95% confidence intervals generated with ggplot2. Differences between groups in terms of size-corrected traits and proportional indices were visualized using violin plots combined with boxplots and statistical annotations. These visualizations were produced using the ggstatsplot package, specifically through functions such as ggbetweenstats, which integrate nonparametric statistical tests, *p* values, and effect sizes within a unified graphical framework. Regression equations and coefficients of determination (R^2^) were extracted from models fitted in stats and incorporated into graphical outputs.

## Results

Geometric morphometric analyses revealed significant variation in head shape among localities, with a strong contribution of geographic structure and allometric effects and no evidence of sexual dimorphism.

**Figure 2:**
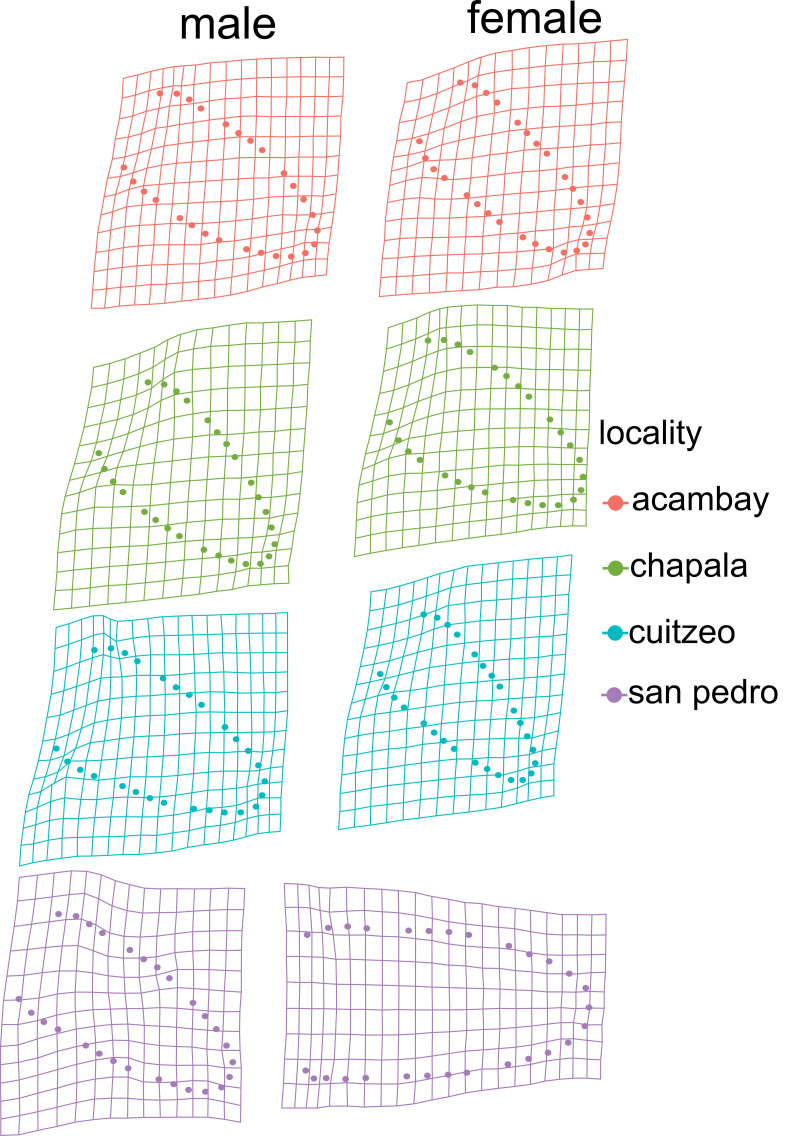
Deformation grids of the head of *T. melanogaster*. Differences in the shape and direction of the vectors in the shape of the head of the snake *T. melanogaster* for each locality.

Procrustes ANOVA indicated that head shape differed significantly among localities, while sex had no significant effect (Table 1, Fig. 2A). Specifically, locality explained a substantial proportion of the shape variation (*F* = 9.94, *Z* = 4.83, *p* = 0.001, *R*^2^ = 0.222), whereas sex had no significant effect (*p* = 0.312, *R*^2^ = 0.008). Centroid size had a significant effect on shape (*F* = 6.59, *Z* = 2.50, *p* = 0.004, *R*^2^ = 0.049), indicating the occurrence of allometric variation. When explicitly testing for allometry, centroid size remained highly significant (*F* = 16.22, *Z* = 3.26, *p* = 0.001, *R*^2^ = 0.122), and locality also remained significant (*F* = 6.16, *Z* = 3.84, *p* = 0.001, *R*^2^ = 0.139); however, the interaction between centroid size and locality was not significant (*p* = 0.380), indicating that allometric trajectories are consistent across populations.

**Table 1:** Results of the Procrustes ANOVA testing the effects of locality, sex, and their interaction on head shape in *Thamnophis melanogaster*. Degrees of freedom (Df), sums of squares (SS), mean squares (MS), proportion of explained variance (Rsq), *F*-values, *Z*-scores, and associated *p*-values (Pr(>F)) are reported. Significant effects (*p* < 0.05) indicate factors contributing to variation in head shape. Residuals represent unexplained variation after accounting for model factors.

	Df	SS	MS	Rsq	*F*	*Z*	Pr(>F)
locality	3	0.0983	0.0327	0.2216	9.4494	4.7139	0.001
sex	1	0.0037	0.0037	0.0084	1.0813	0.4612	0.333
locality:sex	3	0.0120	0.0040	0.0271	1.1561	0.5197	0.318
Residuals	95	0.3297	0.0034	0.7427			
**Total**	102	0.4439					

Principal component analysis of the Procrustes-aligned coordinates revealed structural variation among the populations (Fig. 2B). The first principal component (PC1) captured the main axis of shape variation and was associated with changes in snout elongation and lateral expansion of the cranial region. Morphological trajectories based on mean PC scores indicated directional divergence among populations in morphospace (Fig. 2C). Consistent with these results, hierarchical clustering based on pairwise Procrustes distances showed that Chapala formed a distinct cluster, while the remaining populations were more closely related (Fig. 2D). Heatmap visualization of Procrustes distances confirmed these patterns, with shorter distances occurring among Acambay, Cuitzeo, and San Pedro and greater distances separating Chapala from the other localities (Fig. 2D).

**Figure 3:**
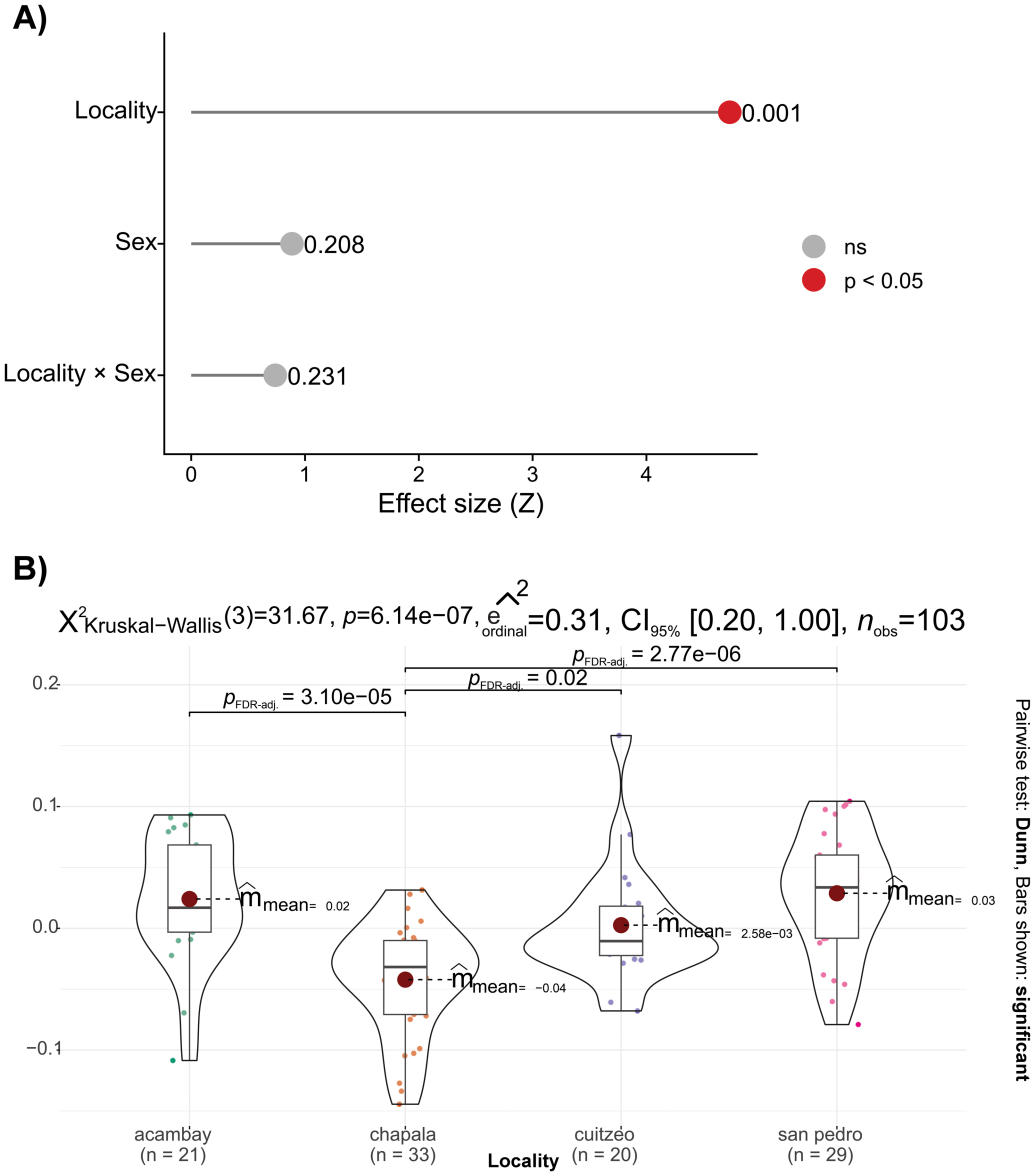
Summary of geometric morphometric analyses of head shape in *Thamnophis melanogaster*. (A) Procrustes ANOVA results showing the contribution of locality, sex, centroid size, and their interaction to shape variation (Rsq). (B) Distribution of PC1 scores across localities, revealing significant differences in head shape among populations (Kruskal–Wallis test).

**Figure 4:**
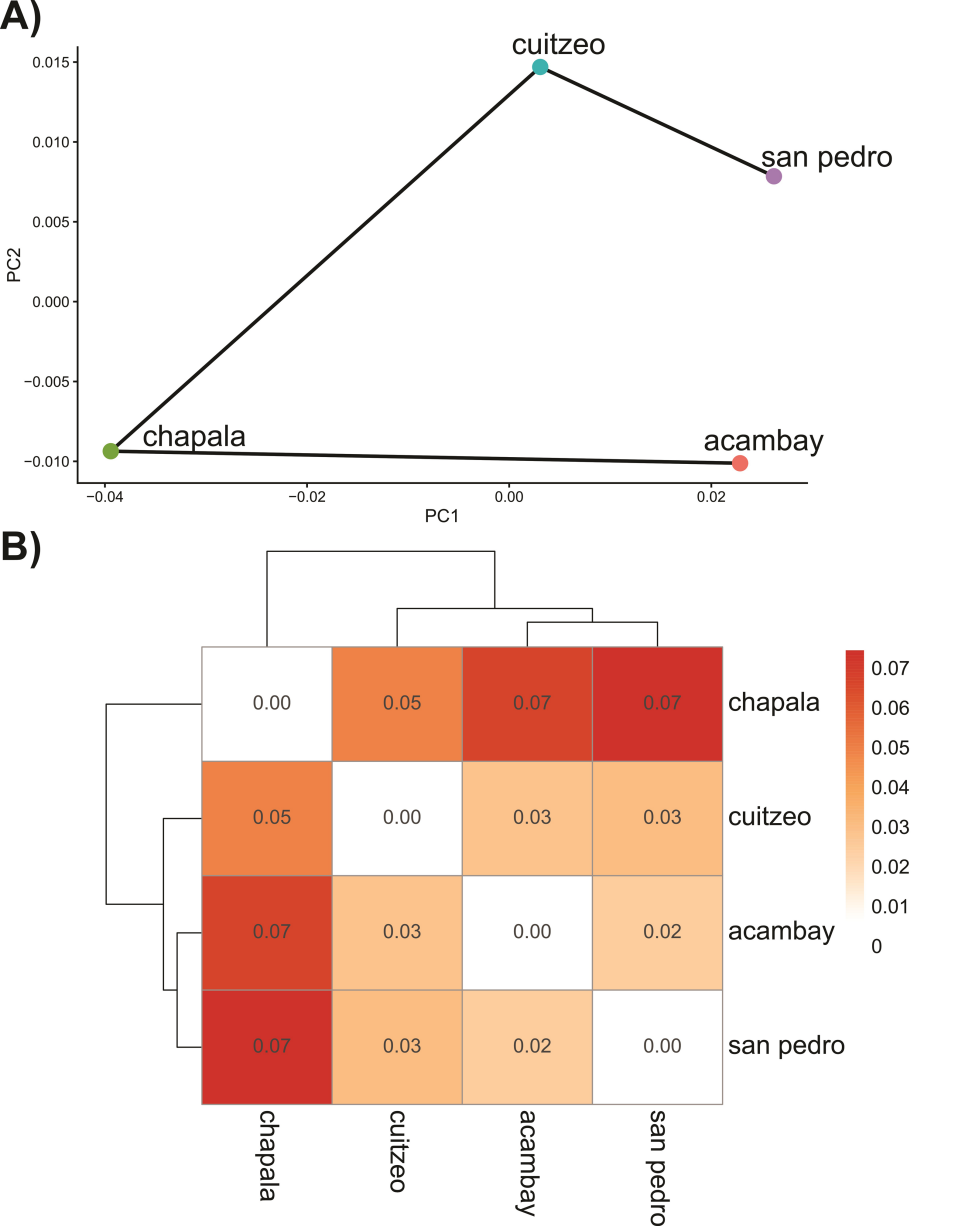
Summary of geometric morphometric analyses of head shape in *Thamnophis melanogaster*. (A) Morphological trajectories based on mean PC scores, illustrating patterns of divergence among populations in morphospace. (B) Heatmap and hierarchical clustering of pairwise Procrustes distances among localities, showing clear separation of the Chapala population from Acambay, Cuitzeo, and San Pedro.

Morphospace visualization revealed partial overlap among the populations, but clear separation patterns were observed, with Chapala occupying a distinct region relative to Acambay, Cuitzeo, and San Pedro (Figs. 3A–3B). Thin-plate spline deformation grids revealed that shape variation among populations was primarily associated with elongation *versus* shortening of the snout and lateral expansion *versus* contraction of the cranial region (Figs. 4A–4B). These patterns were consistent across sexes, reinforcing the lack of sexual dimorphism in head shape.

**Figure 5:**
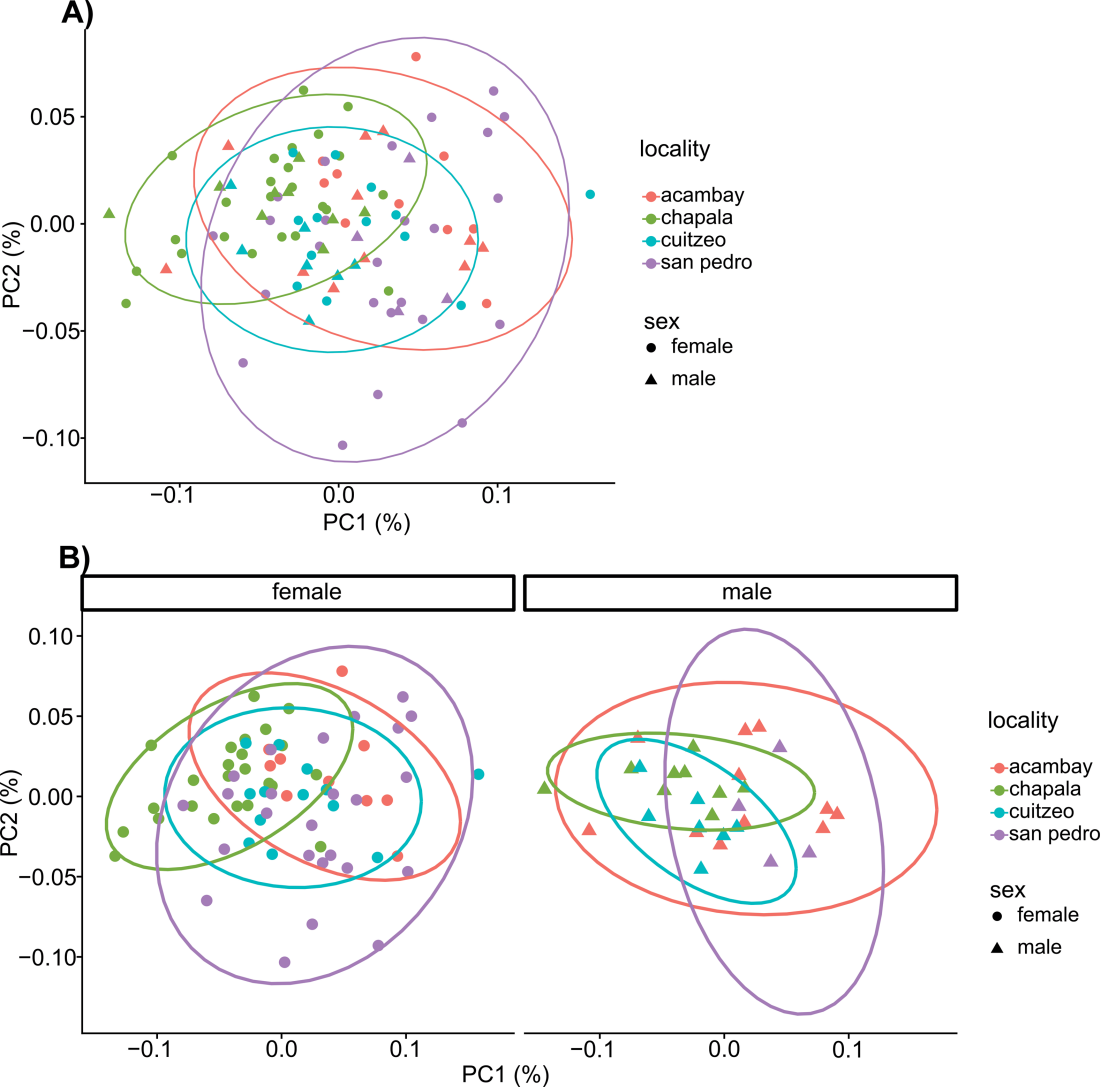
Morphospace and shape variation in head morphology of *Thamnophis melanogaster*. (A) PCA morphospace based on Procrustes-aligned coordinates, showing the distribution of individuals across localities. (B) Morphospace with 95% confidence ellipses and kernel density contours, highlighting differences in dispersion and partial overlap among populations, with Chapala occupying a distinct region.

Because the PC1 scores did not meet parametric assumptions, a Kruskal–Wallis test was performed, revealing significant differences in head shape among localities (*χ*^2^ = 30.13, *df* = 3, *p* = 1.30 × 10^−6^; Fig. 5B). Pairwise Wilcoxon tests with Benjamini–Hochberg correction indicated that Chapala differed significantly from all the other populations (Acambay: *p* = 2.7 × 10^−5^; Cuitzeo: *p* = 0.0027; San Pedro: *p* = 1.0 × 10^−5^), whereas differences among Acambay, Cuitzeo, and San Pedro were not significant or were only marginal (Fig. 5B).

**Figure 6:**
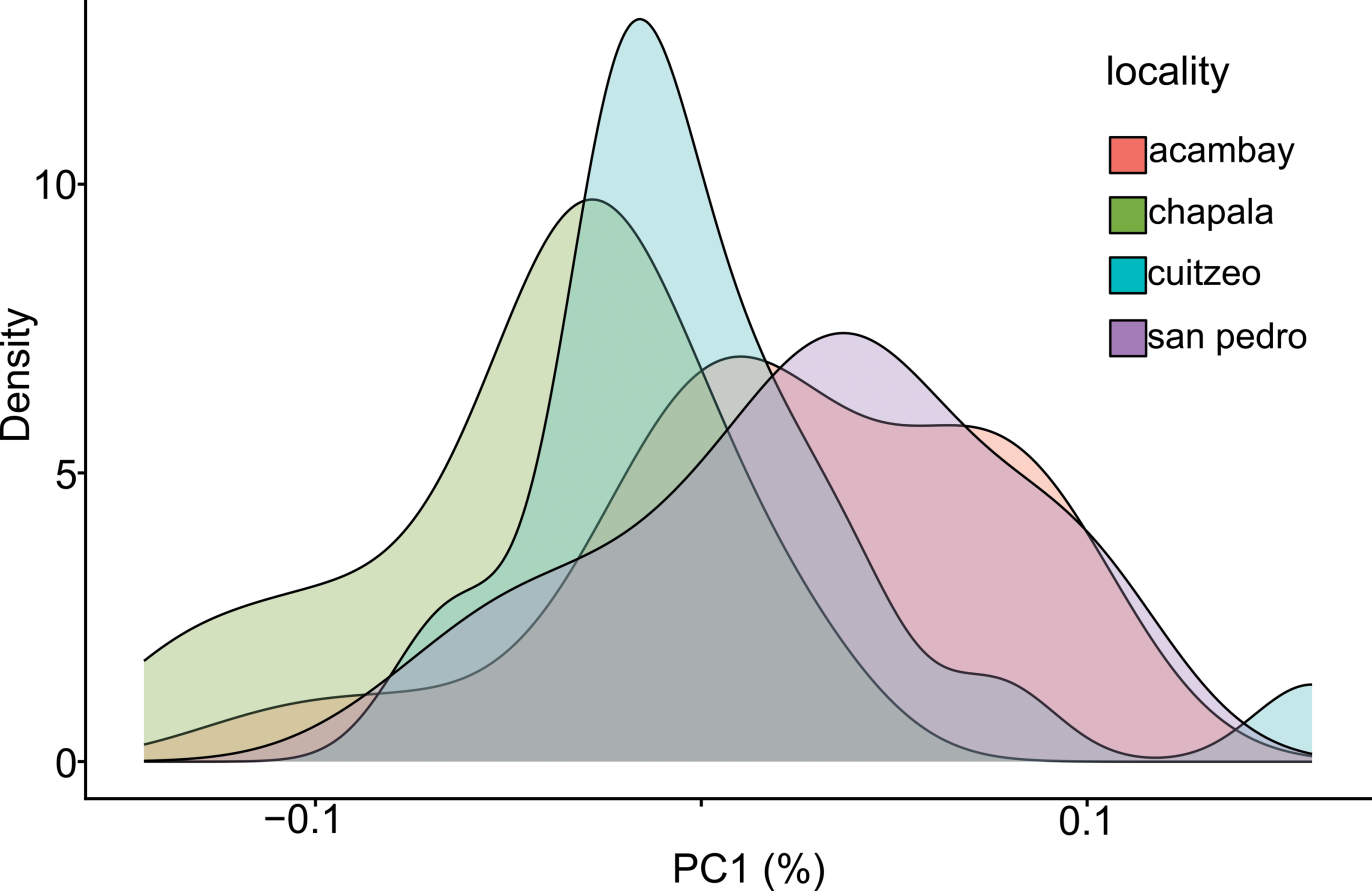
Morphospace and shape variation in head morphology of *Thamnophis melanogaster*. Thin-plate spline deformation grids illustrating shape variation along PC1, emphasizing changes in snout elongation and lateral cranial expansion.

The morphological trajectories among the populations based on the PC scores are shown in Fig. 6, which illustrates directional divergence in morphospace. Additionally, hierarchical clustering and heatmap visualization of pairwise Procrustes distances (Fig. 4B) confirmed that Chapala is morphologically distinct, whereas Acambay, Cuitzeo, and San Pedro exhibit greater similarity.

**Figure 7:**
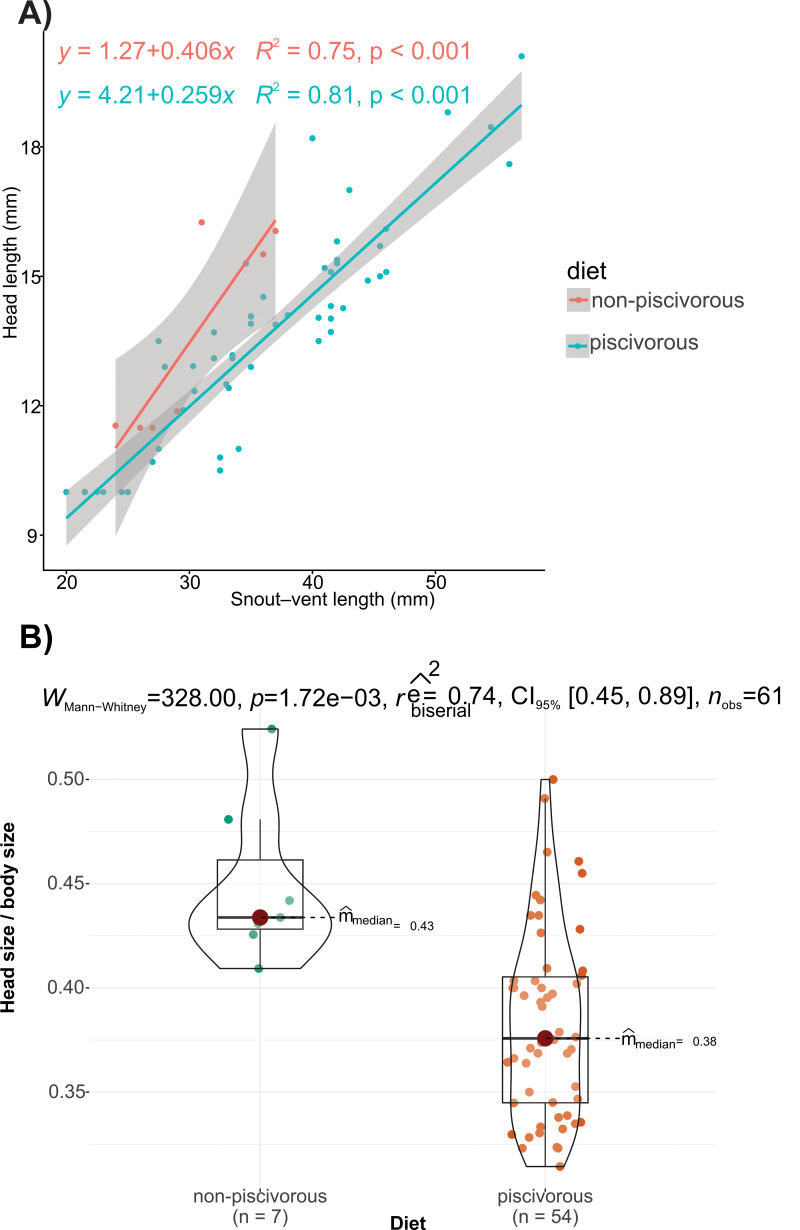
Linear morphometric analyses of head size in relation to diet in *Thamnophis melanogaster*. (A) Relationship between snout–vent length (SVL) and head length, showing similar allometric scaling between piscivorous and non-piscivorous individuals. (B) Relative head size (head length/SVL) differs significantly between dietary groups.

**Figure 8:**
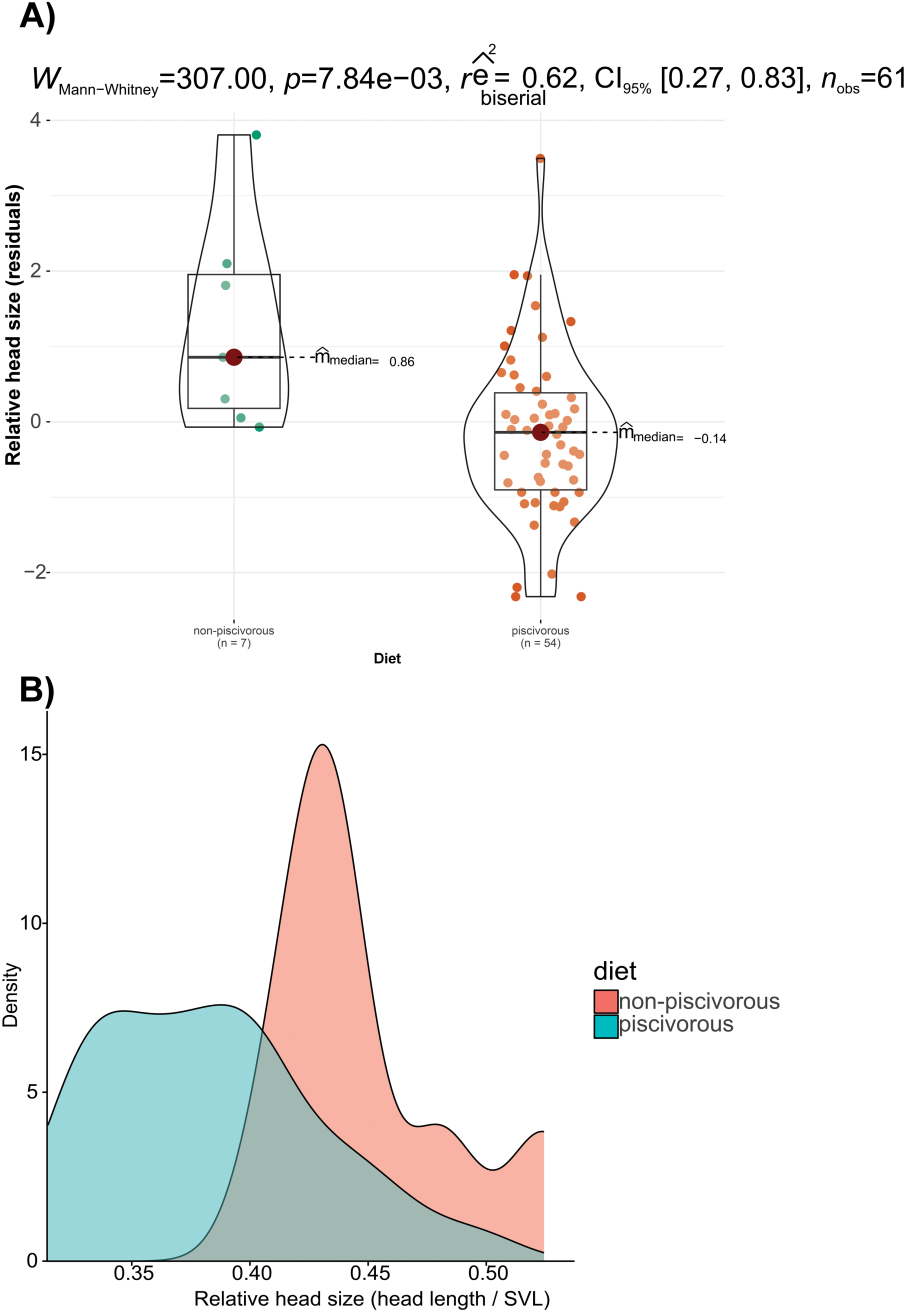
Linear morphometric analyses of head size in relation to diet in *Thamnophis melanogaster*. (A) Size-corrected head size (residuals) also differs between groups, indicating larger relative head size in non-piscivorous individuals. (B) Density distribution of relative head size, illustrating differences in the distribution of head proportions between diets.

Complementary linear morphometric analyses revealed a strong positive relationship between body size (LHC) and head length (estimate = 0.406 ± 0.093, *t* = 4.37, *p* < 0.001, *R*^2^ = 0.805; Fig. 7A), indicating that head size increases predictably with body size. However, diet did not have a significant effect on head size (*p* = 0.314), and the interaction effect between body size and diet was not significant (*p* = 0.125), indicating that allometric scaling was consistent between piscivorous and non-piscivorous individuals (Fig. 7A). Despite the similarity in allometric slopes, size-corrected analyses revealed significant differences between dietary groups. The proportional head size index (head length/LHC) differed significantly between groups (Mann–Whitney U test, *p* = 0.0017; Fig. 7B), suggesting differences in relative head proportions. The residual head size also differed significantly between piscivorous and non-piscivorous individuals (Mann–Whitney U test, *p* = 0.0076; Fig. 8A), indicating that non-piscivorous individuals exhibit relatively larger heads after body size is controlled for. The distribution of relative head size further illustrated these differences between the dietary groups (Fig. 8B).

Taken together, these results indicate that head morphology in *Thamnophis melanogaster* is primarily structured by geographic differences and allometry, while dietary differences do not alter the scaling relationship between head size and body size but do influence relative head proportions.

## Discussion

In this study, we evaluated whether differences in diet (piscivorous *versus* non-piscivorous) influence head morphology in *Thamnophis melanogaster*, under the expectation that piscivorous individuals would exhibit a more elongated head shape associated with hydrodynamic efficiency and prey capture performance. Contrary to this prediction, our results demonstrated that geographic variation, rather than diet, is the primary factor structuring head shape in this species. Geometric morphometric analyses revealed strong and consistent differences among populations, with Chapala representing the most morphologically distinct group, while Acambay, Cuitzeo, and San Pedro showed greater overlap and similarity. This pattern was supported by multiple analytical approaches, including Procrustes ANOVA, PCA morphospace, pairwise comparisons, and clustering analyses, indicating that head shape variation is not random but rather spatially structured. These findings suggest that local environmental conditions, rather than diet alone, play a dominant role in shaping the head morphology of *T. melanogaster*. Geographic variation in morphology is a well-documented phenomenon in snakes and has been associated with environmental heterogeneity, habitat structure, and ecological interactions beyond prey type (Allsteadt et al., 2006; Herrel et al., 2008; Clifton, Chamberlain & Gifford, 2017; Hudry & Herrel, 2025). In this context, populations of *T. melanogaster* are likely exposed to distinct selective pressures related to habitat complexity, hydrodynamic conditions, prey availability, and predator interactions, all of which may influence head morphology (Fabre et al., 2016; Segall et al., 2016; Moon et al., 2019; Deepak, Gower & Cooper, 2023). The clear differentiation of the Chapala population, which was consistently recovered across analyses, suggested that this locality may represent a distinct ecological regime driving morphological divergence. Importantly, we did not detect sexual dimorphism in head shape, which contrasts with patterns commonly reported in snakes, where females typically exhibit larger heads associated with the consumption of larger prey (Shine, 1991; Shine, 1994a; Vincent & Herrel, 2007; Meik et al., 2012). The absence of sexual dimorphism in our study suggests that males and females of *T. melanogaster* may not differ substantially in trophic niche or prey size or that both sexes experience similar ecological constraints. Alternatively, it is possible that sexual differences in this species are expressed as traits other than head shape, such as body size or reproductive traits (Shine, 1994a; Krause, Burghardt & Gillingham, 2003). This result is particularly relevant because it contradicts one of the most common expectations of snake morphology and suggests that sexual divergence in feeding-related traits is not universal across species.

Allometric effects are also an important component of morphological variation. Centroid size had a significant effect on head shape, indicating that shape changes as individuals grow. However, the absence of a significant interaction between size and locality indicates that allometric trajectories are conserved across populations. This finding suggested that although individuals differ in size, the relationship between size and shape follows a common developmental pattern across geographic regions. Such conservation of allometric trajectories may reflect underlying developmental constraints or functional requirements associated with feeding mechanics, limiting the extent to which populations can diverge morphologically (Shine, 1994b; Hudry & Herrel, 2025). Although diet did not explain major differences in head shape, linear and geometric morphometric analyses revealed significant differences in relative head proportions between dietary groups of *T. melanogaster*. Specifically, non-piscivorous individuals exhibited proportionally larger heads after body size was controlled for, as indicated by both residual-based analyses and proportional indices. These findings suggest that diet may influence head morphology at a finer scale, affecting the head morphology proportion rather than the overall shape configuration. Such differences may be functionally relevant, as a larger relative head size could enhance the handling of bulkier or more resistant prey, such as amphibians or invertebrates (Queral-Regil & King, 1998; Meik et al., 2012; Hampton, 2014), although this was not directly tested. In contrast, piscivorous individuals may not require larger head proportions if hydrodynamic efficiency and rapid prey capture are more critical than force generation (Segall et al., 2016). The lack of strong morphological divergence associated with piscivory contrasts with the findings of previous studies suggesting that piscivorous snakes exhibit elongated or specialized head shapes adapted for capturing fish (Herrel et al., 2008; Vincent et al., 2009; Hudry & Herrel, 2025). One possible explanation for this discrepancy is that *T. melanogaster* does not strictly specialize in a single prey type across all populations (Manjarrez, Macías-Garcia & Drummond, 2013) and that dietary variation within populations may reduce the strength of directional selection (Hudry & Herrel, 2025; Wan et al., 2026).

Additionally, aquatic environments impose multiple functional constraints, including hydrodynamic resistance and maneuverability, which may favor intermediate morphologies rather than extreme specialization (Vincent et al., 2009; Segall et al., 2016; Segall, Herrel & Godoy-Diana, 2019). In this sense, head shape in *T. melanogaster* may represent a compromise between competing functional demands, including prey capture, locomotion, and predator avoidance. Furthermore, the observed morphological patterns suggest that factors such as habitat structure and environmental complexity may play more important roles than previously assumed. For example, differences in water clarity, vegetation density, substrate type, or prey behavior could influence how snakes capture and handle prey, thereby affecting morphological adaptation. The strong differentiation of the Chapala population may reflect unique environmental conditions in this locality, potentially including differences in prey communities or habitat characteristics that were not explicitly measured in this study. It is also important to consider that head morphology in snakes is a multifunctional trait. In addition to feeding, the head is involved in defense, sensory perception, and locomotion, particularly in aquatic environments where hydrodynamics play a critical role (Van Wassenbergh et al., 2010; Segall et al., 2020; Pandelis, Grundler & Rabosky, 2023; Hudry & Herrel, 2025). As a result, selection on head shape may be influenced by multiple ecological factors simultaneously, leading to complex patterns of morphological variation that cannot be explained by diet alone. This multifunctionality may partly explain why dietary differences do not translate into clear differences in overall head shape, even when they influence relative proportions. Taken together, our results highlight the importance of integrating multiple analytical approaches when studying morphological variation. While these patterns were supported by nonparametric tests, they should be interpreted with caution given the limited sample size and the absence of strong separation in terms of distributional overlap.

Overall, this study demonstrated that the relationship between snake morphology and diet is more complex than traditionally assumed. Rather than a simple correspondence between prey type and head shape, our findings indicate that multiple ecological and functional factors interact to shape morphological variation. This underscores the need for integrative approaches that consider geography, allometry, and the ecological context when investigating morphological evolution in snakes and other vertebrates. However, future studies incorporating direct measurements of feeding performance or prey mechanics are necessary to confirm these functional interpretations.

## Conclusions

This study demonstrated that head morphology in *Thamnophis melanogaster* is shaped by a complex interplay of geographic variation, allometry, and dietary effects at different levels. Contrary to our initial expectations, head shape variation was primarily structured by differences among localities rather than by diet, with the Chapala population exhibiting the greatest morphological divergence. In contrast, sexual dimorphism in head shape was not detected, suggesting that males and females share similar functional or ecological constraints in this species.

Allometric effects play a significant role in shaping head morphology; however, allometric trajectories are conserved across populations, indicating that developmental and functional constraints may limit divergence in shape. Importantly, although diet did not drive major differences in overall head shape, linear morphometric analyses suggested differences in relative head proportions between piscivorous and non-piscivorous individuals. These results indicate that dietary effects may occur at a finer morphological scale, influencing proportions rather than the global shape configuration.

Overall, our results highlight that morphological variation in *T. melanogaster* cannot be explained by diet alone but instead reflects the combined influence of multiple ecological and evolutionary factors. This underscores the importance of integrating geometric and linear morphometric approaches to fully understand phenotypic variation and suggests that adaptive responses to feeding ecology may be more subtle and context-dependent than traditionally assumed.

## Supplemental Information

10.7717/peerj.21624/supp-1 Supplemental Information 1 Raw data Morphometric data of T. melanogaster

10.7717/peerj.21624/supp-2 Supplemental Information 2 Raw data lineal morphometric data of T. melanogaster used for linear analysis

10.7717/peerj.21624/supp-3 Supplemental Information 3 Procrustes ANOVA Procrustes ANOVA using the geomorph package to evaluate the effects of locality, sex, and their interaction on head shape.

10.7717/peerj.21624/supp-4 Supplemental Information 4 Script for R script used in the R package

## Additional Information and Declarations

### Field Study Permissions

The following information was supplied relating to field study approvals (i.e., approving body and any reference numbers): Secretaria del Medio Ambiente y Recursos Naturales SCPA/DCVS/ 02685 /19) and the ethics committee of the Universidad Autónoma del Estado de México (4047/2016SF, and 4865/2019SF).

### Data Availability

The following information was supplied regarding data availability: The data and code are available in the Supplemental Files.

## References

[ref-1] Adams DC, Collyer ML, Kaliontzopoulou A, Baken EK (2021). Geomorph: software for geometric morphometric analyses. https://cran.r-project.org/package=geomorph.

[ref-2] Adams D, Collyer M, Kaliontzopoulou A, Baken E (2026). Geomorph: software for geometric morphometric analyses. https://cran.r-project.org/package=geomorph.

[ref-3] Alfaro ME (2002). Forward attack modes of aquatic feeding garter snakes. Functional Ecology.

[ref-4] Allsteadt J, Savitzky AH, Petersen CE, Naik DN (2006). Geographic variation in the morphology of Crotalus horridus (Serpentes: Viperidae). Herpetological Monographs.

[ref-5] American Society of Ichthyologists and Herpetologists (ASIH) (2004). Guidelines for use of live amphibians and reptiles in field and laboratory research. https://ssarherps.org/wp-content/uploads/2014/07/guidelinesherpsresearch2004.pdf.

[ref-6] Baken E, Collyer M, Kaliontzopoulou A, Adams D (2021). geomorph v4.0 and gmShiny: enhanced analytics and a new graphical interface for a comprehensive morphometric experience. Methods in Ecology and Evolution.

[ref-7] Clifton IT, Chamberlain JD, Gifford ME (2017). Patterns of morphological variation following colonization of a novel prey environment. Journal of Zoology.

[ref-8] Collyer M, Adams D (2018). RRPP: an R package for fitting linear models to high-dimensional data using residual randomization. Methods in Ecology and Evolution.

[ref-9] Collyer M, Adams D (2024). RRPP: linear model evaluation with randomized residuals in a permutation procedure. https://cran.r-project.org/package=RRPP.

[ref-10] Deepak V, Gower DJ, Cooper N (2023). Diet and habit explain head-shape convergences in natricine snakes. Journal of Evolutionary Biology.

[ref-11] Dwyer CM, Kaiser H (1997). Relationship between skull form and prey selection in the thamnophiine snake genera *Nerodia* and *Regina*. Journal of Herpetology.

[ref-12] Fabre AC, Bickford D, Segall M, Herrel A (2016). The impact of diet, habitat use, and behavior on head shape evolution in homalopsid snakes. Biological Journal of the Linnean Society.

[ref-13] Fox J, Weisberg S (2019). An R companion to applied regression, third edition.

[ref-14] Gibbons JW, Dorcas ME (2004). North American Watersnakes: a natural history.

[ref-15] González-Fernández A, Manjarrez J, García-Vázquez U, D’Addario M, Sunny A (2018). Present and future ecological niche modeling of garter snake species from the Trans-Mexican Volcanic Belt. PeerJ.

[ref-16] Hampton PM (2011). Comparison of cranial form and function in association with diet in natricine snakes. Journal of Morphology.

[ref-17] Hampton PM (2014). Allometry of skull morphology, gape size and ingestion performance in the banded watersnake (*Nerodia fasciata*) feeding on two types of prey. Journal of Experimental Biology.

[ref-18] Herrel A, Vincent SE, Alfaro ME, Van Wassenbergh S, Vanhooydonck B, Irschick DJ (2008). Morphological convergence as a consequence of extreme functional demands: examples from the feeding system of natricine snakes. Journal of Evolutionary Biology.

[ref-19] Hudry D, Herrel A (2025). Divergent paths, convergent heads: morphological adaptation of head shape to habitat use and diet in snakes. Journal of Morphology.

[ref-20] INEGI (2013). México en cifras: información nacional, por entidad federativa y municipios. https://www.inegi.org.mx/app/buscador/default.html?q=acambay.

[ref-21] Klaczko J, Sherratt E, Setz EZ (2016). Are diet preferences associated with skull shape diversification in xenodontine snakes?. PLOS ONE.

[ref-22] Krause MA, Burghardt GM, Gillingham JC (2003). Body size plasticity and local variation of relative head and body size sexual dimorphism in garter snakes (*Thamnophis sirtalis*). Journal of Zoology.

[ref-23] Madsen T, Shine R (1993). Phenotypic plasticity in body sizes and sexual size dimorphism in European grass snakes. Evolution.

[ref-24] Manjarrez J, Macías-Garcia C, Drummond H (2013). Variation in the diet of the Mexican black-bellied garter snake *Thamnophis melanogaster*: importance of prey availability and snake body size. Journal of Herpetology.

[ref-25] Manjarrez J, Macías-Garcia C, Drummond H (2017). Morphological convergence in a Mexican garter snake associated with the ingestion of a novel prey. Ecology and Evolution.

[ref-26] Manjarrez J, Macias-Garcia C, Drummond H (2020). Congenital feeding response to a novel prey in a Mexican gartersnake. PeerJ.

[ref-27] Manjarrez J, Pacheco-Tinoco M, Venegas-Barrera CS (2017). Intraspecific variation in the diet of the Mexican garter snake *Thamnophis eques*. PeerJ.

[ref-28] Marko PB (2005). An intraspecific comparative analysis of character divergence between sympatric species. Evolution.

[ref-29] Meik JM, Setser K, Mociño Deloya E, Lawing AM (2012). Sexual differences in head form and diet in a population of Mexican lance-headed rattlesnakes, Crotalus Polystictus. Biological Journal of the Linnean Society.

[ref-30] Moon BR, Penning DA, Segall M, Herrel A, Bels V, Whishaw IQ (2019). Feeding in snakes: form, function, and evolution of the feeding system. Feeding in Vertebrates: evolution, morphology, behavior, biomechanics.

[ref-31] Mori A, Vincent SE (2008). An integrative approach to specialization: relationships among feeding morphology, mechanics, behavior, performance and diet in two syntopic snakes. Journal of Zoology.

[ref-32] Nair A (2016). IMP: interactive model performance evaluation. https://CRAN.R-project.org/package=IMP.

[ref-33] Pandelis GG, Grundler MC, Rabosky DL (2023). Ecological correlates of cranial evolution in the megaradiation of dipsadine snakes. BMC Ecology and Evolution.

[ref-34] Patil I (2021). Visualizations with statistical details: the ‘ggstatsplot’ approach. Journal of Open Source Software.

[ref-35] Perkins M, Eason P (2019). The relationship of head morphology and diet among three sympatric watersnake species. Amphibia-Reptilia.

[ref-36] Queral-Regil A, King RB (1998). Evidence for phenotypic plasticity in snake body size and relative head dimensions in response to amount and size of prey. Copeia.

[ref-37] R Core Team (2024). R: a language and environment for statistical computing. https://www.R-project.org/.

[ref-38] Rivera G (2008). Ecomorphological variation in shell shape of the freshwater turtle *Pseudemys concinna* inhabiting different aquatic flow regimes. Integrative and Comparative Biology.

[ref-39] Rossman DA, Ford NB, Seigel RA, Rossman DA, Ford NB, Seigel RA (1996). Species accounts. The garter snakes: evolution and ecology.

[ref-40] Segall M, Cornette R, Fabre AC, Godoy-Diana R, Herrel A (2016). Does aquatic foraging impact head shape evolution in snakes?. Proceedings of the Royal Society B: Biological Sciences.

[ref-41] Segall M, Cornette R, Godoy-Diana R, Herrel A (2020). Exploring the functional meaning of head shape disparity in aquatic snakes. Ecology & Evolution.

[ref-42] Segall M, Herrel A, Godoy-Diana R (2019). Hydrodynamics of frontal striking in aquatic snakes: drag, added mass, and the possible consequences for prey capture success. Bioinspiration & Biomimetics.

[ref-43] Shine R (1991). Intersexual dietary divergence and the evolution of sexual dimorphism in snakes. The American Naturalist.

[ref-44] Shine R (1994a). Sexual size dimorphism in snakes revisited. Copeia.

[ref-45] Shine R (1994b). Allometric patterns in the ecology of Australian snakes. Copeia.

[ref-46] Valencia-Flores E, Venegas-Barrera CS, Fajardo V, Manjarrez J (2019). Microgeographic variation in body condition of three Mexican garter snakes in central Mexico. PeerJ.

[ref-47] Van Wassenbergh S, Brecko J, Aerts P, Stouten I, Vanheusden G, Camps A, Van Damme R, Herrel A (2010). Hydrodynamic constraints on prey-capture performance in forward-striking snakes. Journal of the Royal Society Interface.

[ref-48] Vincent SE, Brandley MC, Herrel A, Alfaro ME (2009). Convergence in trophic morphology and feeding performance among piscivorous natricine snakes. Journal of Evolutionary Biology.

[ref-49] Vincent SE, Herrel A (2007). Functional and ecological correlates of ecologically based dimorphisms in squamate reptiles. Integrative and Comparative Biology.

[ref-50] Wan X, She W, Liu C, Huang G, Li G, Zhang H, Liu J, Wei F (2026). Linking ecological and biogeographical traits to body length and dietary breadth in snakes. Integrative Zoology.

[ref-51] Wickham H (2016). ggplot2: elegant graphics for data analysis.

[ref-52] Wickham H, François R, Henry L, Müller K, Vaughan D (2026). dplyr: a grammar of data manipulation. https://CRAN.R-project.org/package=dplyr.

[ref-53] Wickham H, Vaughan D, Girlich M (2025). tidyr: tidy messy data. https://CRAN.R-project.org/package=tidyr.

